# Multisystem Myotilinopathy, including Myopathy and Left Ventricular Noncompaction, due to the *MYOT* Variant c.179C>T

**DOI:** 10.1155/2020/5128069

**Published:** 2020-05-13

**Authors:** Josef Finsterer, Claudia Stöllberger, Matthias Hasun, Korbinian Riedhammer, Mathias Wagner

**Affiliations:** ^1^Krankenanstalt Rudolfstiftung, Messerli Institute, Vienna, Austria; ^2^2nd Medical Department with Cardiology and Intensive Care Medicine, Krankenanstalt Rudolfstiftung, Vienna, Austria; ^3^Institute of Human Genetics, Germany; ^4^Department of Nephrology, Klinikum Rechts der Isar, School of Medicine, Technical University of Munich, Munich, Germany

## Abstract

Left ventricular hypertrabeculation/noncompaction is a myocardial abnormality of unknown etiology/pathogenesis, which is frequently associated with neuromuscular disorders or chromosomal defects. LVHT in association with a *MYOT* mutation has not been reported. The patient is a 72-year-old male with a history of strabismus in childhood, asymptomatic creatine-kinase elevation since age 42 years, slowly progressive lower limb weakness since age 60 years, slowly progressive dysarthria and dysphagia since age 62 years, and recurrent episodes of arthralgias and myalgias since age 71 years. He also had arterial hypertension, diverticulosis, hyperlipidemia, coronary heart disease, and a hiatal hernia with reflux esophagitis. Clinical exam revealed mild quadruparesis and proximal wasting of the legs. Whole exome sequencing revealed a known variant in the *MYOT* gene. Muscle biopsy, previously assessed as inclusion body myopathy, was compatible with the genotype after revision. Cardiologic work-up revealed a left anterior hemiblock, mild myocardial thickening, and noncompaction. This case shows that myotilinopathy may manifest as a multisystem disease, including noncompaction.

## 1. Introduction

Left ventricular hypertrabeculation (LVHT), also known as left ventricular noncompaction (LVNC), is a myocardial anomaly characterised by hypertrabeculation of the left ventricular apex [1]. It is frequently associated with monogenic disease or chromosomal defects, but a causal relation has not been established [1]. If neuromuscular disorders (NMDs) are systematically screened for LVHT, up to 80% of the NMD patients show LVHT on echocardiography or cardiac MRI [1, 2]. Though LVHT has been associated with a number of NMDs, myotilinopathy (myopathy due to a variant in the *MYOT* gene most frequently manifesting as limb girdle muscular dystrophy type 1A (LGMD1A)) has not been reported in association with LVHT. Additionally, long-term, slowly progressive creatine-kinase (CK) elevation, which may be the first sign in LGMDs, has been only rarely reported as a manifestation of myotilinopathy. Here, we present a patient in whom a pathogenic variant in the *MYOT* gene was detected by whole exome sequencing (WES), who presented with late-onset, slowly progressive myopathy and LVHT.

## 2. Case Report

The patient is a 72-year-old Caucasian male, height 186 cm, weight 94 kg, and with a previous history of strabism since childhood resulting in impaired vision of the right eye, asymptomatic CK elevation first detected at age 42 years, arterial hypertension since age 57 years, diverticulosis of the sigma detected at age 57 years, slowly progressive weakness of the lower limbs since the age of 60 years, slowly progressive dysarthria and dysphagia since age 62 years, and hyperlipidemia since the age of 62 years. At age 66 years, a drug-eluting stent (DES) was implanted into the left anterior descending (LAD) coronary artery. Despite stenting, the patient experienced a non-ST elevation myocardial infarction (NSTEMI) due to coronary two-vessel disease 6 months later. The history was further positive for hiatal hernia with reflux disease since age 68 years and for resection of a left transverse colon polyp at age 66 years. Five months prior to the last presentation, the patient experienced recurrent episodes of arthralgias and myalgias together with chills. Three months prior to the last presentation, he was able to climb stairs upwards only with a rail and used two sticks for walking on an even floor. The patient was regularly taking acetyl-salicylic acid, ticagrelor, esomeprazole, ramipril, nebivolol, and evolocumab since he did not tolerate statins. The family history was positive for CK elevation in his son who refused to undergo diagnostic work-up for myotilinopathy.

A clinical neurologic exam at age 72 years revealed weakness for hip extension (right M5-, left M4), for knee extension (right M5-, left M4+), for knee flexion (M4+ bilaterally), and for foot extension (right M4+, left M5-), diffuse wasting of the lower limb muscles with proximal predominance, hypoesthesia of the lateral left lower limb, absent tendon reflexes on the lower limbs, mild edema of the feet and ankles, bilateral contractures of the Achilles tendons with incipient tiptoe walking, and missing pulses of the dorsalis pedis arteries bilaterally.

CK was elevated to values between 200 and 500 U/L. There was recurrent elevation of CK-MB and troponin, but proBNP was either normal or only mildly elevated. Nerve conduction studies revealed symmetric, axonal polyneuropathy. Muscle MRI revealed marked wasting and replacement of the muscle tissue by fat in the proximal limb muscles, most pronounced in the lateral, intermedius, and medial vastus muscles; the semimembranosus and semitendinosus muscles; and the adductors but largely sparing the gracilis and the sartorius muscles. Muscle biopsy from the left lateral vastus muscle at age 60 years revealed fiber size variation, numerous atrophic fibers, regenerating fibers, fiber necrosis, some ragged-red and COX-negative fibers, and large rimmed vacuoles with APP-, tau-, and ubiquitin-positive depositions. In the vicinity of these vacuoles, tubulofilamentous inclusions were seen on electron microscopy. This is why initially, inclusion body myopathy was suspected [3].

**Figure 1:**
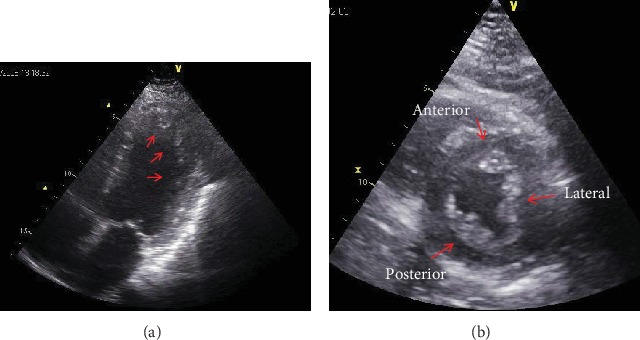
Echocardiographic apical 4-chamber view showing left ventricular hypertrabeculation/noncompaction affecting the apex of the left ventricle (a). Echocardiographic short axis view of the left ventricle with hypertrabeculation/noncompaction affecting the anterior, lateral, and posterior walls (b).

**Figure 2:**
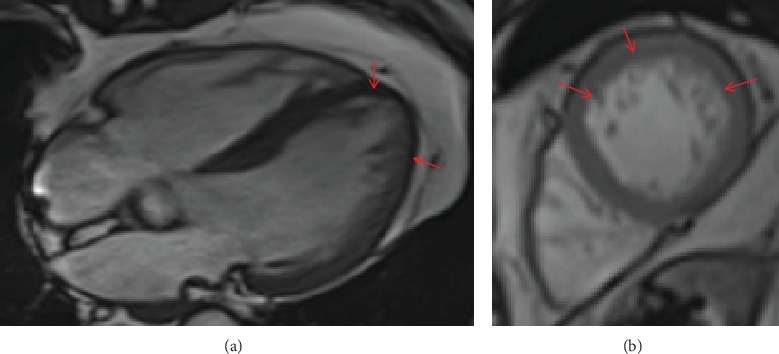
Cardiac MRI (3T, true fisp) showing left ventricular hypertrabeculation/noncompaction in the end-diastolic 4-chamber view (a) and in the apical short axis view (b).

ECG revealed a left anterior hemiblock. Holter ECG and 24 h blood pressure monitoring were normal. Transthoracic echocardiography showed normal systolic function but thickening of the left ventricular myocardium and LVHT (Figure 1). LVHT was confirmed by cardiac MRI at age 71 years (Figure 2). A CT scan of the brain was normal. MRI of the brain at age 61 years revealed spot-like, T2-hyperintense lesions of the white matter and some lacunas in the left crus cerebrum. Carotid ultrasound showed only mild atherosclerosis. Videocinematography only revealed impaired motility of the esophagus. Impedance manometry revealed ineffective motility of the bolus along the esophagus. WES at age 79 years revealed the known missense variant c.179C>T in the *MYOT* gene.

## 3. Discussion

The presented patient is interesting for several aspects. First, the variant c.179C>T has been only occasionally described in patients with myotilinopathy. Generally, *MYOT* variants manifest phenotypically as myofibrillar myopathy [4, 5], spheroid-body myopathy [6], pseudohypertrophy and muscle stiffness [7], distal myopathy [8, 9], or as LGMD1A [10, 11]. Since there is a broad overlap between these phenotypes, they are summarised under the term myotilinopathies [12]. The variant found in the index patient has been previously reported in a patient with myofibrillar myopathy [11], in a French family with late onset nonspecific myopathy [13], and in a patient with severe proximal muscular dystrophy [10]. Additionally, the variant has been reported in two British patients with late onset myofibrillar myopathy [14]. The first homozygous (recessive) *MYOT* variant was detected in a German patient with progressive myofibrillar myopathy [4]. The variant c.179C>T is listed in ClinVar as pathogenic.

Second, the initial manifestation was long-term CK elevation, which has not been described in myotilinopathy before. However, CK elevation in the absence of any other clinical manifestation is often the first sign in limb girdle muscular dystrophies.

**Figure 3:**
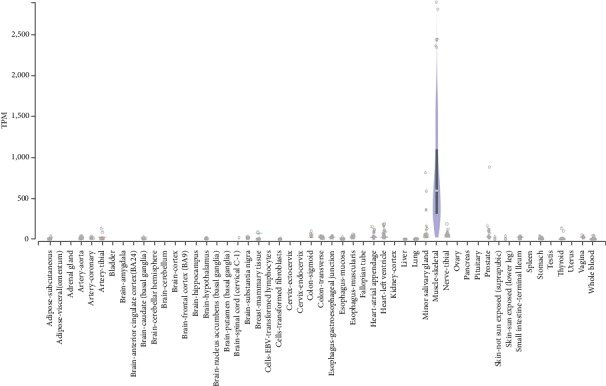
Gene expression for MYOT (ENSG00000120729.5).

Third, the patient manifested with a multisystem disease, affecting not only the muscles but also the brain (dysarthria, dysphagia), heart (myocardial thickening, LVHT), and peripheral nerves (axonal polyneuropathy). A multisystem disease due to *MYOT* variants affecting the skeletal muscles as well as other organs (heart, peripheral nerves) has been previously reported although myotilin is predominantly expressed in the skeletal muscle (Figure 3). Phenotypes of previously reported patients with myotilinopathy [11] showed similarities with the index patient. In accordance with the index patient, patients carrying other *MYOT* variants manifested in the peripheral nerves as polyneuropathy [11], as cardiomyopathy [11], or as dysphagia or dysarthria [15]. In a French family with myotilinopathy [13], muscle biopsy showed large rimmed vacuoles similar to those found in the index patient. Whether hiatal hernia and diverticulosis were related to the *MYOT* variant remains speculative, but affection of the autonomous innervation is more likely than affection of the smooth muscle cells as myotilin is not expressed in smooth muscle cells. Tightened heel cords are, on the contrary, frequently seen in patients with myotilinopathy [15]. Not only patients with late onset and slow progression of the phenotype have been reported, but also patients with early onset [15] and rapid progression [16]. Atherosclerosis, manifesting as a coronary heart disease and arterial occlusive disease, was regarded as unrelated to the *MYOT* variant.

Arrhythmias or LVHT have not been reported in association with *MYOT* variants. LVHT in the index patient represents a unique phenotypic feature but nonetheless needs to be recognised since it may strongly influence the outcome of these patients. LVHT is well-known for complications such as cardioembolism, heart failure, or ventricular arrhythmias potentially leading to sudden cardiac death [1]. Cardioembolism can be prevented by oral anticoagulation, systolic dysfunction may respond to heart failure therapy, and arrhythmias may respond to antiarrhythmics or device implantation. LVHT has not been reported in any other limb girdle muscular dystrophy before.

In conclusion, this case shows that myotilinopathy may manifest with LVHT and not only in the skeletal muscle but also in the brain, heart, and peripheral nerves. Patients with myotilinopathy should be investigated by not only the neurologist but also the cardiologist.

## Additional Points

*Highlight*. MYOT mutations may manifest with left ventricular noncompaction. Myotilinopathies may manifest with mild, long-term creatine-kinase elevation. Myotilinopathies may manifest as a multisystem disease.
